# *Domitiuslusitanicus* (Araneae, Nesticidae) - an umbrella species for the conservation of troglobionts in the Estremenho Karst Massif, Portugal

**DOI:** 10.3897/BDJ.12.e124103

**Published:** 2024-06-27

**Authors:** Tomás R. L. Alves, Ana Sofia P. S. Reboleira

**Affiliations:** 1 Centre for Ecology, Evolution and Environmental Changes (cE3c) & CHANGE - Global Change and Sustainability Institute, and Departamento de Biologia Animal, Faculdade de Ciências, Universidade de Lisboa, Lisbon, Portugal Centre for Ecology, Evolution and Environmental Changes (cE3c) & CHANGE - Global Change and Sustainability Institute, and Departamento de Biologia Animal, Faculdade de Ciências, Universidade de Lisboa Lisbon Portugal; 2 Museu Nacional de História Natural e da Ciência, Universidade de Lisboa, Rua da Escola Politécnica, Lisbon, Portugal Museu Nacional de História Natural e da Ciência, Universidade de Lisboa, Rua da Escola Politécnica Lisbon Portugal

## Abstract

**Background:**

*Domitiuslusitanicus* (Fage, 1931) is a troglobiont spider, endemic from caves in the largest karst massif in Portugal, the Estremenho. It was the first described cave-adapted species from Portugal, but the male of the species was only described in 1988.

**New information:**

Over the last two decades, the knowledge on the distribution of *D.lusitanicus* increased significantly. We assess the conservation status of *D.lusitanicus*, providing new information on its extent of occurrence and the anthropogenic threats and present a IUCN Red List profile. *D.lusitanicus* faces various anthropogenic threats, such as habitat loss, agriculture, pollution and tourism impacts. Despite a large part of its distribution is included in a Natural Park, it expands outside of the areas deemed for protection in the Natura 2000 network. This species has the widest spread distribution of all troglobionts in the Estremenho Massif; therefore, it may be used as an umbrella species for the protection of other cave-adapted species of invertebrates of the massif.

## Introduction

Cave-adapted communities have high conservationist interest (Mammola et al. 2019), they are habitat specialists with high endemicity patterns, reduced population numbers and low fertility traits (Deharveng et al. 2024). Cave fauna face direct anthropogenic threats, such as groundwater contamination, habitat destruction due to quarrying activities and excessive cave visitation (Castaño-Sánchez et al. 2020).

Nesticidae is a small family of spiders with a worldwide distribution that includes 16 genera and 289 described species (World Spider Catalog 2023). Seven genera and 56 species are known from Europe, distributed from the Iberian Peninsula to the Caucasus and the Ural Mountains (Ribera and Dimitrov 2023). Most of these European species are cave dwellers and many of them are troglobionts, i.e. cave-adapted species. Due to these habitat preferences, many nesticids present small and confined distributions, as in cave habitats (Ribera and Dimitrov 2023). *Domitius* Ribera, 2018 is an independent evolutionary lineage of nesticids, a sister group to the clade formed by *Kryptonesticus* Pavlek & Ribera, 2017, *Carpathonesticus* Lehtinen & Saaristo, 1980 and *Nesticus* Thorell, 1869 (Ribera 2018).

*Domitiuslusitanicus* (Fage, 1931) was described in 1931 under the genus *Nesticus* by Louis Fage, based on female specimens. Male specimens were only described 67 years later (Ribera 1988). It is an endemic troglobiont species of Portugal, only recorded in caves of the Estremenho Karst Massif, making it a priority species for conservation actions. This species was recently profiled for conservation (Branco et al. 2019), but a wide array of new information on its distribution and threats made it necessary to update and develop its conservation profile. Additionally, it is the most widespread troglobiont species in its karst area (Reboleira 2012) and can, therefore, be used as an umbrella species (Roberge and Angelstam 2004) for subterranean invertebrate biodiversity endemic to its Estremenho Massif. We assessed the conservation profile of *D.lusitanicus* based on recently-collected data.

## Material and methods

Caves of the Estremenho Karst Massif, located in central Portugal, were sampled by direct search for the past two decades, under permits of the Instituto de Conservação da Natureza e das Florestas. The specimens were sorted and identified to species level through dissection and microscopy. *D.lusitanicus* is also recorded from "Algar das Aranhas" Cave in the Estremenho Karst Massif (Reboleira 2012), based on specimens from museum collections, but the location of this cave is currently unknown, therefore, excluded from the analysis.

The extent of occurrence (EOO) and area of occupancy (AOO) were calculated using the Geospatial Conservation Assessment Tool (GeoCAT) with an approximation to the standard IUCN 2 km × 2 km cells (4 km^2^). Maps were created in the open-source software QGIS v.3.22.6 (QGIS Development Team 2009), with the hypsometry of Portugal and the total protected area bound by “Parque Natural da Serra de Aire e Candeeiros” layers (DGT 2023, ICNF 2023).

Threats were identified *in situ*, complemented with literature surveys and satellite images provided by Google Earth software. These threats, as well as habitat classification and conservation measures were assigned, based on the IUCN Red List criteria.

## Species Conservation Profiles

### Domitius lusitanicus

#### Species information

Scientific name: Domitiuslusitanicus

Species authority: (Fage, 1931)

Kingdom: Animalia

Phylum: Arthropoda

Class: Arachnida

Order: Araneae

Family: Nesticidae

Taxonomic notes: Distinct paracymbium shape and details in the palpal bulbs in male spiders, as well as clear morphological differences in the epigyne and vulva in the females (Ribera 1988).

**Figure 1.**
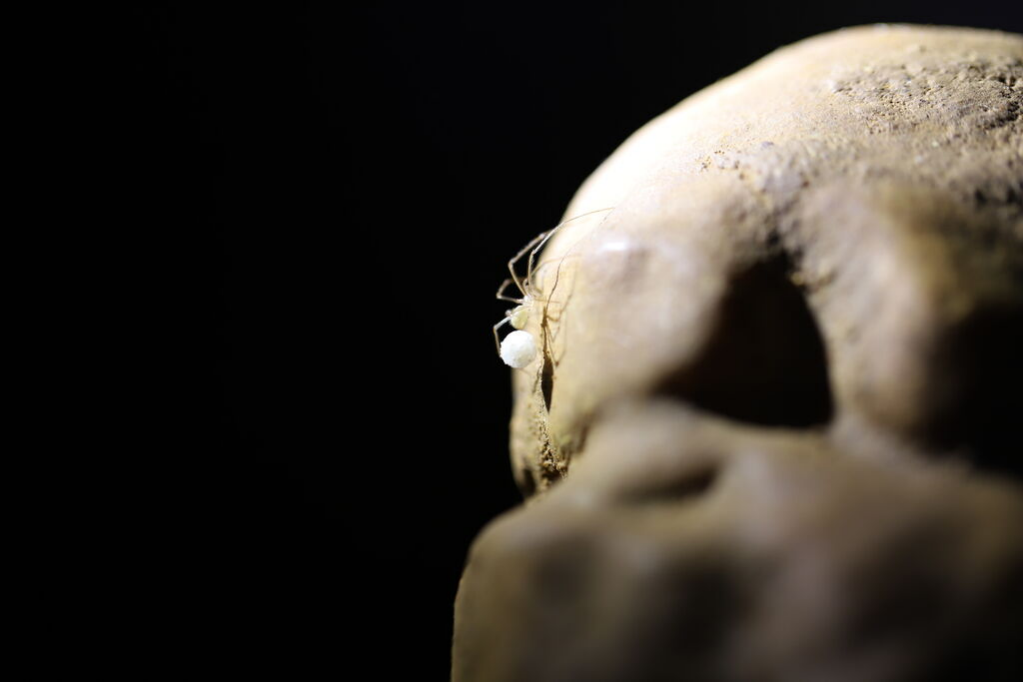
*Domitiuslusitanicus* (Fage, 1931), female carrying an egg sac in the Ervideira Cave, Aljubarrota Platform, Portugal.

**Figure 2.**
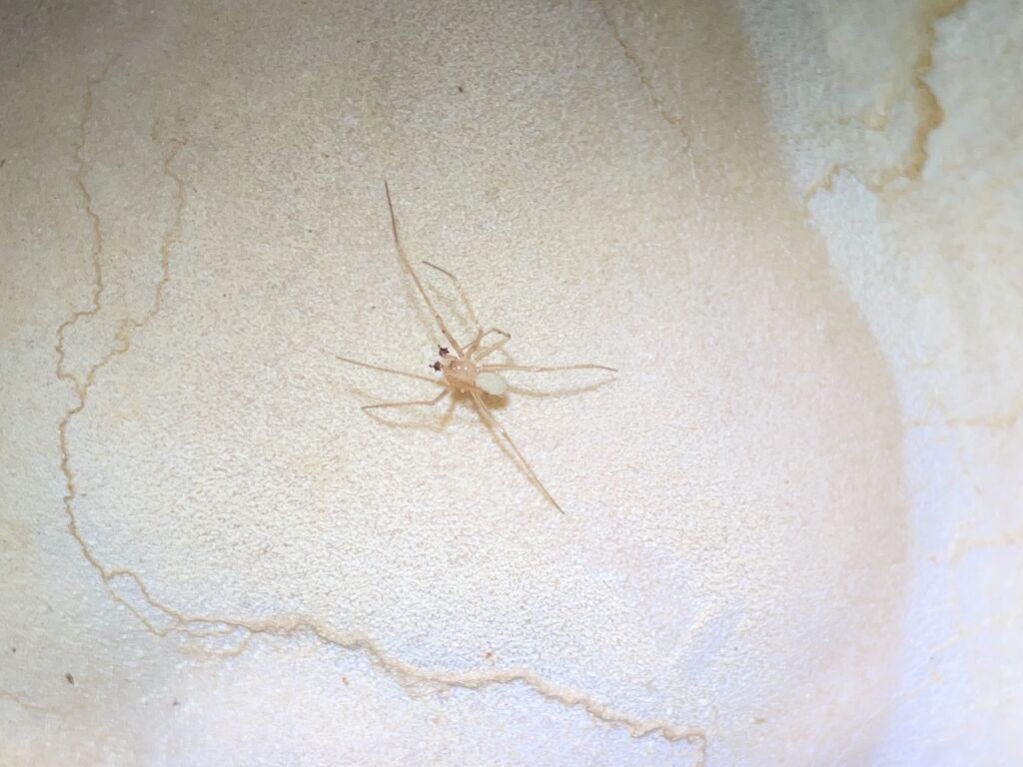
*Domitiuslusitanicus* (Fage, 1931), male walking on the cave wall in the Santuário Cave, Mira de Aire, Portugal.

Figure(s) or Photo(s): Figs 1, 2

Region for assessment: Europe

#### Geographic range

Biogeographic realm: Palearctic

Countries: Portugal

**Figure 3.**
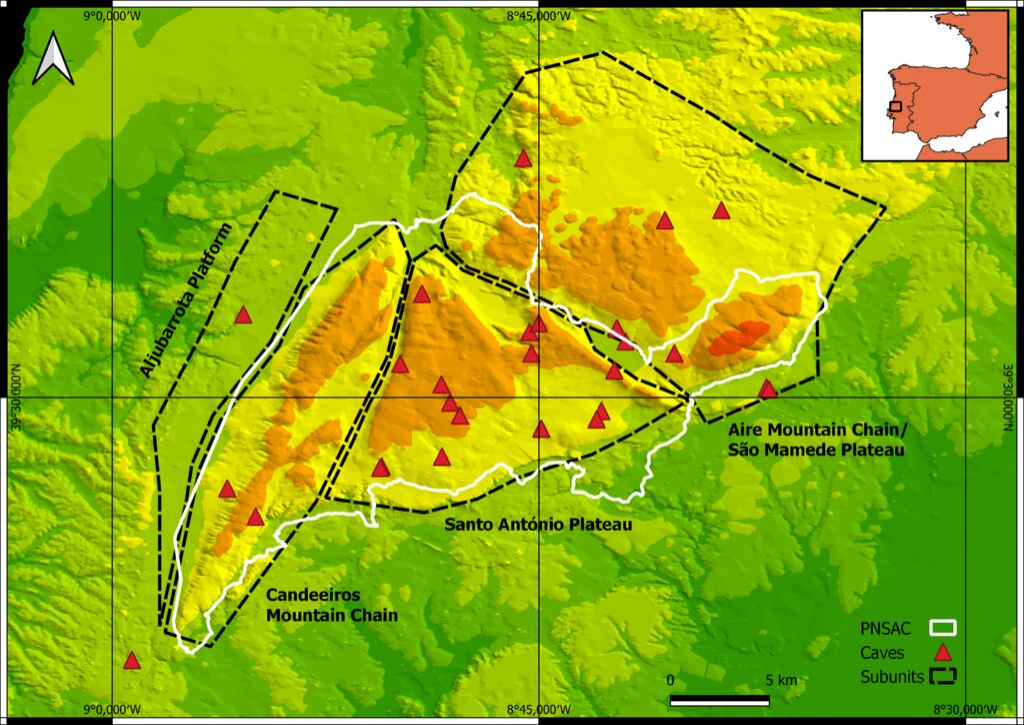
Distribution of *Domitiuslusitanicus* (Fage, 1931). Serras de Aire e Candeeiros Natural Park (PNSAC) delimited by the white line.

Map of records (image): Fig. 3

Map of records (Google Earth): Suppl. material 1

Basis of EOO and AOO: Known habitat extent

Basis (narrative): The extent of occurrence (EOO) is 534.790 km^2^ and the area of occupancy (AOO) is 88.0 km^2^.

Min Elevation/Depth (m): 105

Max Elevation/Depth (m): 495

Range description: *Domitiuslusitanicus* was recorded from 28 caves distributed along the Estremenho Karst Massif across its four main subunits (Fig. 3): São Mamede Plateau/Aire Mountain Chain, Santo António Plateau, Candeeiros Mountain Chain and Aljubarrota Plateau. In the São Mamede Plateau/Aire Mountain Chain subunit, it was recorded in: Buraco Roto Cave, Lapa da Salgada Cave, Algar do Burro Cave, Mira de Aire Cave (Moinhos Velhos Cave), Contenda Cave (with underwater connection to Moinhos Velhos Cave), Santuário Cave, Almonda Cave and Algar da Lomba Cave. In the Santo António Plateau subunit, it was recorded in: Lapa da Ovelha Cave, Morcegos Cave, Lapa da Chã de Cima Cave, Algar da Pena Cave, Pinheiro Cave, Algar da Cheira Cave, Algar da Arroteia Cave, Algar da Manga Larga Cave, Algar do Zé de Braga Cave, Algar das Marradinhas II Cave, Algar do Chou Jorge Cave, Lapa dos Pocilgões Cave, Algar das Gralhas I Cave, Algar das Gralhas VII Cave, Algar do Ladoeiro Cave and Algar do João Malhão Cave. In Candeeiros Mountain Chain subunit, it was recorded in: Alcobertas Cave, Senhora da Luz Cave and Algar do Vale da Pena Cave (= Algar dos Ursos) Cave. In the Aljubarrota Plateau subunit, it was recorded in Ervideira Cave.

#### Extent of occurrence

EOO (km2): 534.79

Trend: Unknown

Causes ceased?: Unknown

Causes understood?: Unknown

Causes reversible?: Unknown

Extreme fluctuations?: Unknown

#### Area of occupancy

Trend: Unknown

Causes ceased?: Unknown

Causes understood?: Unknown

Causes reversible?: Unknown

Extreme fluctuations?: Unknown

AOO (km2): 88

#### Locations

Number of locations: 28

Justification for number of locations: *Domitiuslusitanicus* occurs in 28 caves of the Estremenho Karst Massif.

Trend: Unknown

Extreme fluctuations?: Unknown

#### Population

Number of individuals: Unknown

Trend: Unknown

Causes ceased?: Unknown

Causes understood?: Unknown

Causes reversible?: Unknown

Extreme fluctuations?: Unknown

#### Subpopulations

Trend: Unknown

Extreme fluctuations?: Unknown

Severe fragmentation?: Unknown

#### Habitat

System: Terrestrial

Habitat specialist: Yes

Habitat (narrative): The caves are located at an elevation ranging from 105 to 495 m above sea level. The Buraco Roto Cave (Fátima) limits the distribution at the North, while the Senhora da Luz Cave (Rio Maior) is the southern and westernmost locality of the distribution. The Almonda Cave (Torres Novas) represents currently the easternmost locality for the species' distribution.

Trend in extent, area or quality?: Decline (inferred)

##### Habitat

Habitat importance: Major Importance

Habitats: 7.1. Caves and Subterranean Habitats (non-aquatic) - Caves

#### Ecology

Size: 2.9-3.5 mm

Generation length (yr): 1

Dependency of single sp?: Unknown

Ecology and traits (narrative): *Domitiuslusitanicus* is a troglobiont species, it is blind, depigmented and has elongated legs. This species is usually found in the walls of caves of Estremenho Karst Massif with high relative humidity, after the twilight zone throughout the deepest parts. We have observed that, without disturbance, the females stay in the same web for more than year. Males are more rare and are found normally walking throught the caves.

#### Threats

**Figure 4.**
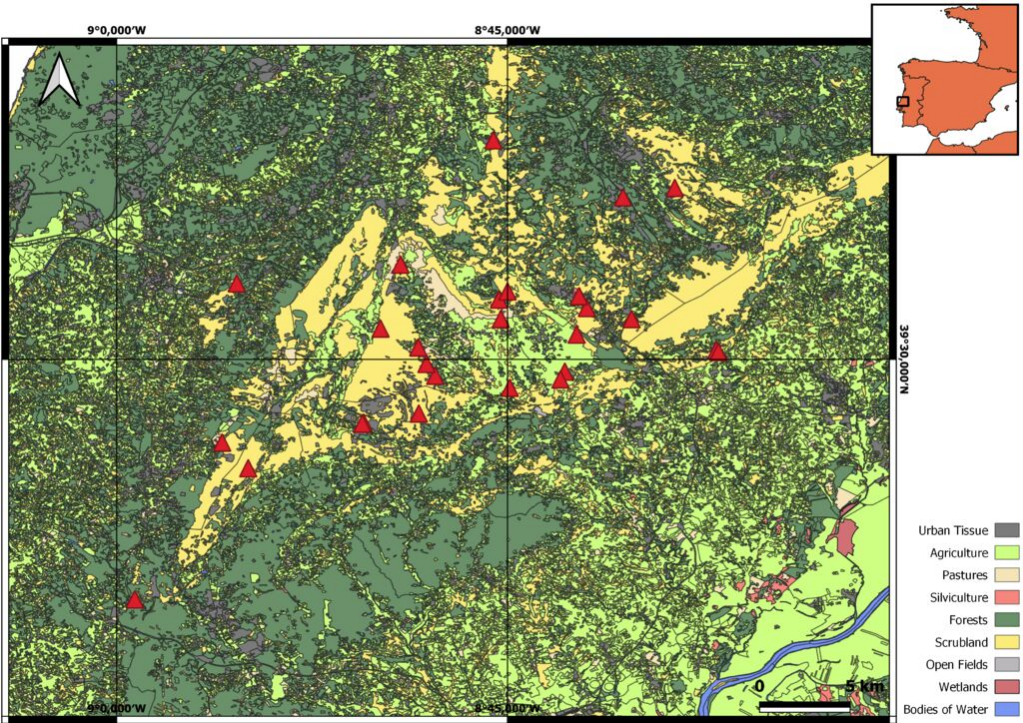
Map of the land use in the Estremenho Karst Massif. Red triangles represent the caves where *Domitiuslusitanicus* is distributed.

Justification for threats: The overall area of distribution of *Domitiuslusitanicus* is highly disturbed by human activities (Fig. 4). Almonda Cave and Pinheiro Cave are located 50 m from a factory that extracts water from the spring feed by its subterranean river and 420 m from a village, which has many agricultural fields. Algar do Ladoeiro Cave entrance is 840 m from the closest urbanisation. The subterranean streams in Mira de Aire and Contenda caves (connected underwater) have a high input of sewage from the surface and are located below the Village of Mira de Aire; therefore, both caves are extremely contaminated by pollutants produced at the surface (Reboleira 2007). The Mira de Aire Cave is the largest show cave of Portugal with around 140,000 visitors per year and, since the 1960s, has had much infrastructure built for touristic exploitation, with a 300 m long show cave section (Reboleira et al. 2015). Santuário Cave is also located in the Mira de Aire Village, with a parking lot three metres from its entrance. Alcobertas Cave is located 640 m from a field of windmills, 1 km from a quarry, 850 m from agricultural lands and 690 m from the nearest village. It has been exploited for touristic activities since the 1970s and an artificial secondary entrance has been opened, drastically changing the climatic conditions (Reboleira 2007, Reboleira et al. 2009). Algar do Vale da Pena Cave is in an abandoned quarry, 700 m from the closest village. Algar do Burro Cave is located 500 m from a quarry, 560 m from the A1 highway and 600 m from the closest village. Lapa da Chã de Cima Cave is located 500 m from a quarry. Algar do Zé de Braga Cave is located below intensive agricultural olive production, where the use of pesticides and fertilisers is prevalent. Pesticides and fertilisers are known to have a harmful effect on subterranean biota and easily infiltrate into underground habitats (Reboleira et al. 2013b, Castaño-Sánchez et al. 2020). Algar do Pena Cave hosts an interpretation centre, has received visitations since 1997 (Popova 2022) and is located 300 m from a quarry. Algar das Gralhas I Cave and Algar das Gralhas VII Cave are located 125 m and 168 m from the same quarry, respectively. Algar das Marradinhas II Cave is located 1.5 km from the nearest village in an area of olive and cattle production (Reboleira et al. 2022). Algar da Arroteia is located 112 m from the closest urbanisation and 1.3 km from a quarry. Lapa da Salgada Cave is located 270 m from a road used by large trucks to transport produce from warehouses 600 m away and is also located 1 km away from the closest town. Lapa dos Pocilgões Cave is located 270 m from the nearest urbanisation and 545 m from a quarry. Algar do Chou-Jorge Cave is located 500 m from the nearest village. Algar da Manga Larga Cave is located 300 m from the nearest village. Algar da Cheira Cave is located 460 m from the nearest urbanisation and has cattle maintenance on the surface. Algar da Lomba Cave develops below the A1 highway, it is located 30 m from the closest urbanisation, 350 m from a village and 2 km from a wind farm. Buraco Roto Cave is located 125 m from the closest village and 450 m from a quarry. Senhora da Luz Cave is located 35 m from the closest urbanisation, 760 m from a quarry and 1.9 km from the A15 highway. Ervideira Cave stands between two quarries, 715 m and 990 m away and is located 590 m and 750 m from the nearest villages. Lapa da Ovelha Cave is located 900 m from the nearest village. Morcegos Cave is located 390 m from the nearest urbanisation and 410 m from a quarry. Algar do João Malhão Cave is located 700 m from the nearest urbanisation.

##### Threats

Threat type: Ongoing

Threats: 1.1. Residential & commercial development - Housing & urban areas1.2. Residential & commercial development - Commercial & industrial areas2.1. Agriculture & aquaculture - Annual & perennial non-timber crops3.2. Energy production & mining - Mining & quarrying3.3. Energy production & mining - Renewable energy6.1. Human intrusions & disturbance - Recreational activities

#### Conservation

Justification for conservation actions: The Contenda and Mira de Aire caves are located below the Village of Mira de Aire and infiltration of sewage is observed underground. To prevent wastewater run-off into subterranean galleries and groundwaters, measures to improve sewage treatment are necessary. Almonda Cave is protected due to its archaeological heritage and has been classified as a Property of Public Interest (IIP) since 1993 (Hoffmann et al. 2013). Despite that, the archaeological protection figures do not contemplate protection of cave-adapted fauna, so it is urgent to set protective measures appropriate to this species. Of the 28 caves, only 21 are protected under legislation by the “Rede Natura 2000” (Directive H 1992, ICNB 2000). All caves with this endemic species should have a protected area delimited at the surface that includes all drainage area of the caves. The caves Algar do Burro, Almonda, Pinheiro, Buraco Roto, Ervideira, Lapa da Salgada and Senhora da Luz should be included in protection figures, as they are currently located out of protected areas. It is recommended to develop a conservation plan for *Domitiuslusitanicus*, encompassing studies to better understand the extent of this species distribution, population size, abundance, life cycle and ecology of the species. Moreover, environmental education about the importance of subterranean habitats and species is fundamental for long-term conservation, specifically targeted mainly for children in local schools and workers from the municipalities included in the distribution area of this species.

##### Conservation actions

Conservation action type: Needed

Conservation actions: 1.1. Land/water protection - Site/area protection1.2. Land/water protection - Resource & habitat protection2.1. Land/water management - Site/area management4. Education & awareness5.1.3. Law & policy - Legislation - Sub-national level

## Discussion

Troglobionts as *Domitiuslusitanicus* have reduced extent of occurrence (EOO) and area of occupancy (AOO), exhibiting highly endemic patterns of distribution (Reboleira et al. 2011). These species have reduced populations, inhabit habitats with specific environmental conditions and are very sensitive to anthropogenic pressure, such as climate change and pollution, being fundamental for global biodiversity conservation (Castaño-Sánchez et al. 2020). Therefore, they constitute a unique biodiversity heritage that is on the of extinction (Mammola et al. 2019). The risk of extinction can be avoided by implementing protection figures for species and habitats specific for troglobionts (Wynne et al. 2021).

Extensive sampling over the last two decades demonstrated that *Domitiuslusitanicus* has a wide distribution across the Estremenho Karst Massif and towards the Aljubarrota Platform, where it is confined (Reboleira 2012). This increased the area of occupancy and extent of occurrence of *D.lusitanicus*, from the previous 199.936 km^2^ (Branco et al. 2019) to 534.790 km^2^. A large area in this karst is threatened by anthropogenic activities, such as pollution, habitat degradation, tourism, alteration of the surface natural cover, urbanism, industries and agriculture (Reboleira 2012, Reboleira et al. 2013b, Reboleira et al. 2022, Reboleira and Eusébio 2021). Although many of these threats have been identified previously, no specific conservation measures have been put in place (Reboleira et al. 2011, Reboleira et al. 2022, Reboleira and Eusébio 2021). Additionally, *D.lusitanicus* is distributed in several caves left out of protected areas.

*Domitiuslusitanicus* is the most widespread troglobiont in the Estremenho Massif, distributed in all caves known to harbour troglobiont species in the massif (Reboleira et al. 2013a, Reboleira et al. 2022, Reboleira and Enghoff 2018, Reboleira and Eusébio 2021, Reboleira and Eusébio 2023). Therefore, it is a key species for biological conservation in this region. It can act as umbrella species for all other cave-adapted species of the Estremenho Massif, because the protection measures concerning this species will ensure the conservation of all other cave-adapted species in this area and, by extension, of subterranean ecosystems in general.

This study can contribute to territory management and planning and to aid in delineating protection strategies for cave-adapted species of the Estremenho Karst Massif in Portugal. We offer detailed information about the distribution *Domitiuslusitanicus* and the current threats to its survival. It is also essential to improve the knowledge on its life cycle, population size, functional ecology, extent of subterranean distribution and sensitivity to disturbance. This information is fundamental to raise awareness through school programmes and national campaigns on the threats that subterranean fauna and habitats face, which will allow for the implementation of conservation efforts to prevent the extinction of endemic species.

Discussion

## Supplementary Material

218473C9-7DEA-52E0-B72F-10B8BC0FD44D 10.3897/BDJ.12.e124103.suppl1 Supplementary material 1 Distribution of Domitiuslusitanicus Data type occurrences Brief description Distribution of the cave-adapted spider *Domitiuslusitanicus* (Fage, 1931). File: oo_1034735.kml https://binary.pensoft.net/file/1034735 Alves T. & Reboleira A.S.P.S.
